# Circularly assembled genome sequences of *Asticcacaulis* sp. strain MM231 and *Brevundimonas subvibrioides* MM232, isolated from a pond at Bielefeld University campus

**DOI:** 10.1128/mra.00699-24

**Published:** 2024-09-27

**Authors:** Bianca Laker, Tim Schulze, Mara Reis, Sophia-Maria Bormann, Gianluca Radke, Levin Joe Klages, Tobias Busche, Lutz Wobbe, Andrea Bräutigam, Marion Eisenhut

**Affiliations:** 1 Computational Biology, Faculty of Biology, Bielefeld University, Bielefeld, Germany; 2 Center for Biotechnology (CeBiTec), Bielefeld University, Bielefeld, Germany; 3 Biology, Bielefeld University, Bielefeld, Germany; 4 Microbial Genomics and Biotechnology, Center for Biotechnology (CeBiTec), Bielefeld University, Bielefeld, Germany; 5 Bielefeld University, Medical School East Westphalia-Lippe, Bielefeld University, Bielefeld, Germany; University of Maryland School of Medicine, Baltimore, Maryland, USA

**Keywords:** genomes, aquatic habitat

## Abstract

Photosynthetic organisms often interact with heterotrophic microbes. We here report the complete genome sequences of the bacterial strains *Asticcacaulis* sp. MM231 and *Brevundimonas subvibrioides* MM232. Both bacteria were co-isolated from a single green colony originating from an aquatic sample taken from a pond at the Bielefeld University campus.

## ANNOUNCEMENT

In aquatic systems, photosynthetic organisms live together with bacteria, as they exude sugars that serve as a carbon source for the heterotrophic bacteria. For the genus *Brevundimonas,* living in symbiotic consortia with microalgae was shown (1), while for a member of the *Asticcacaulis,* an interaction with the aquatic plant duckweed was observed (2). To enhance the knowledge of bacteria co-living with photosynthetic aquatic autotrophs, we cultivated waterborne samples on a medium without a carbon source and isolated DNA from the green, chlorophyll-containing colonies.

The aquatic sample was collected from the surface water of a pond located at the Bielefeld University campus, Germany (52°2′6.4″N 8°29′43.3″E) and centrifuged. The cell pellet was resuspended in fresh 0.9% NaCl solution. Dilutions were plated on solidified BG11 medium (3) and incubated on a windowsill at 22°C for 7 days. A single green colony was picked and streaked on an agar medium. After 7 days of growth, DNA was isolated from the streaked biological material on the plate with the NucleoSpin Microbial DNA kit (Macherey-Nagel, Düren, Germany), and RNA was digested. The ligation sequencing kit V14 [SQK-LSK114; Oxford Nanopore Technologies (ONT), Oxford, UK] following the manufacturer’s protocol without DNA shearing and size selection was used for the preparation of sequencing libraries. One R10.4.1 flow cell was run for 21 hours on a GridION (ONT). The super-accurate base-calling model from MinKNOW v23.07.5 (ONT) was used for base-calling producing 390,379 raw reads with an *N*_50_ of 16,082 bp. All programs were run with default parameters unless otherwise specified. Adapters were trimmed with Porechop v0.2.4 (4), and the reads were used in a blastn search (BLAST 2.14.0+) (5) with parameters “-evalue 0.0001 -max_hsps 1” against NCBI’s prokaryotic RefSeq genome database (6). Reads matching the genus *Asticcacaulis* or *Brevundimonas* were assembled with Canu v2.2 (7) using a genome size of 5 or 3.2 Mbp, respectively. The assemblies resulted in a single genome contig each, with two (MM231) or one (MM232) additional sequence (Table 1). Yet, these additional sequences were not circularly assembled. Contigs were polished two times with racon v1.5.0 (8) and minimap v2.26 with parameter “-ax map-ont” (9) and two times with Medaka v1.8.0 applying “-m r1041_e82_400bps_sup_v4.2.0” (ONT). Overlapping ends were removed with Berokka v0.2.3 (https://github.com/tseemann/berokka), and sequences were rotated such that they start with *dnaA* using Circlator v1.5.5 (10). Phylogenetic classification was performed with the Type (Strain) Genome Server (TYGS) (11). Genome completeness of the circular genomic contigs was validated each with benchmarking universal single-copy orthologs (BUSCO) v5.5.0 setting “--mode geno” (12). Genes were annotated with Prokka v1.14.6 (13). Relevant statistics for the assemblies are reported in Table 1.

**TABLE 1:** Sequencing and assembly statistics for *Asticcacaulis* sp. strain MM231 and *Brevundimonas subvibrioides* MM232^*a*^

	Finding for	
Parameter	*Asticcacaulis* sp. strain MM231	*Brevundimonas subvibrioides* MM232
Genomic sequence		
Length (bp)	3,458,694	3,281,735
GC content (%)	59.00	68.40
Genome coverage (×)	43.65	38.45
Gene annotation		
Total no. of genes	3,432	3,247
No. of protein-coding genes	3,377	3,183
No. of rRNAs	6	6
No. of tRNAs	49	59
BUSCO results (%)		
Complete	94.6	96.8
Single copy	94.4	96.3
Duplicated	0.2	0.5
Fragmented	4.4	2.1
Missing	1.0	1.1
Other contigs		
No. of sequences	2	1
Length (bp)	(a) 618,632 (b) 570,284	17,430
GC content (%)	(a) 57.30 (b) 56.80	56.80
Coverage (×)	(a) 41.86 (b) 36.52	10.70
Total no. of genes	(a) 500 (b) 441	10
No. of protein-coding genes	(a) 499 (b) 435	10
No. of rRNAs	(a) 0 (b) 3	0
No. of tRNAs	(a) 1 (b) 3	0

^*a*^ The used database and the number of searched BUSCOs were alphaproteobacteria_odb10 with 432 BUSCO genes. For the other sequences in the *Asticcacaulis* assembly, (a) refers to sequence “MM231_p1” and (b) to “MM231_p2.”

The genome sequences of *Asticcacaulis* sp. strain MM231 and *Brevundimonas subvibrioides* MM232 can be classified as complete. Both were circularly assembled, and they have a high number of complete BUSCO genes. According to TYGS, the here represented *Asticcacaulis* sp. has *Asticcacaulis benevestitus* DSM 16100 as the closest relative (GenBank accession number GCA_000376105.1), and the *Brevundimonas* sp. has *Brevundimonas subvibrioides* ATCC 15264 as the closest relative (GenBank accession number GCA_000144605.1).

## Data Availability

The MM231 and MM232 assemblies, gene annotations, and reads are available in GenBank/ENA under BioProject accession number PRJEB75201. The GenBank/ENA accession number for the MM231 raw reads is ERR13189148 and GCA_964186625.1 for the MM231 assembly and annotation. The GenBank/ENA accession number for the MM232 raw reads is ERR12945363 and GCA_964034995.1 for the MM232 assembly and annotation.
